# Review of Several Books

**Published:** 1891-01

**Authors:** 


### Book Notices.

ESSENTIALS OF GYNECOLOGY, arranged in the form of questions and answers prepared specially for students of medicine, by Edwin B. Cragin, M. D., Gynecologist to the Roosevelt hospital. With 58 illustrations. Philadelphia: W. B. Saunders, publisher.

This is a neat little book of 190 pages. It contains a great deal for the space. Its aim is to furnish a ready means for a hasty review of the subject before the student comes up for examination. It is as good a work as one could find of the kind.

B.

---

ESSENTIALS OF EXAMINATION OF THE URINE, by Lawrence Wolff, M. D., Demonstrator of Chemistry in the Jefferson Medical College. Philadelphia: W. B. Saunders, publisher. Price, 75 cents.

The directions given in this book seem to be thorough. It contains only 62 pages. The examinations are made chemically and microscopical, with a special view to the practical. It is a neat, well gotten up little book. B.

---

THE MEDICAL ANNUAL AND PRACTITIONERS' INDEX. A work of reference for medical practitioners. Eighth year. 1890. New York: E. B. Treat & Co. Price, $2.75; pp., 570.

This book is a little better gotten up than former editions of the Annual. The binding is more substantial, and the pages a trifle larger, though the type is still rather small for the average reader. It is a summary of the year's work in medicine, and is valuable for reference. Send to the publishers for a copy, and then keep up your subscription annually thereafter. It is a good book. B.

